# Personalisation schemes in social care: are they growing social and health inequalities?

**DOI:** 10.1186/s12889-019-7168-4

**Published:** 2019-06-24

**Authors:** Eleanor Malbon, Gemma Carey, Ariella Meltzer

**Affiliations:** Centre for Social Impact, UNSW, Kensington, Australia

**Keywords:** Personalisation, health inequalities, individual funding, social inequality

## Abstract

**Background:**

The connection between choice, control and health is well established in the literature on the social determinants of health, which includes choice and control of vital health and social services. However, even in the context of universal health and social care schemes, the ability to exercise choice and control can be distributed unequally. This paper uses the case of the Australian National Disability Insurance Scheme (NDIS) to examine these issues. The NDIS is a major policy reform based on an international trend towards personalisation in social care. It aims to increase choice and control over services and supports for people who have or acquire a permanent disability, thereby boosting citizen empowerment and improving health and social outcomes.

**Methods:**

The research is a structured review of empirical evidence on the administration and outcomes of the NDIS to identify how social factors constrain or enable the ability of individuals to exercise choice within personalised care schemes.

**Results:**

We show how social determinants of health at the individual level can collide with the complexity of policy delivery systems to entrench health inequalities.

**Conclusion:**

Many social policy reforms internationally focus on improving empowerment through enabling choice and control. However, if administrative systems do not take account of existing structural inequities, then such schemes are likely to entrench or grow social inequality. Our research indicates that more attention must be given to the design of policy delivery systems for personalisation schemes to ensure health equity.

## Main text

Poor, or unequal, access to health and social services is a major determinant of social inequalities in health [20, 45, 46, 75]. Differential access, or quality, of services can occur because of geographical, economic and cultural reasons [75]. Even if the same services are offered, their take up and benefit can be unequal – ‘impartiality’ in services (i.e. where everyone receives the same service) is not the same as universal access [8]. These issues are most famously captured by Hart’s ‘inverse care law’ [25]. Social determinants of health research has also established that choice and control over ones lives is an important driver of health outcomes [20, 45, 46, 76]. This includes choice and control over the health and social services.

These public health debates have been echoed in social policy, with growing concern for citizen control and empowerment has been mirrored in debates in social policy, with a push towards person centered approaches to social care, referred to as personalisation [22]. As just one of a variety of ‘particularist’ approaches to social policy [8], which aim to put the individual at the centre of decision making, the goal of personalisation is to provide services which cater to a diversity of social and cultural needs, and enable people to make a choice about the services and supports they receive from governments [69]. Proponents argue that such approaches encourage citizen empowerment, resulting in better outcomes [70]. Evidence on the social determinants of health suggests that putting people in control of their lives, including services and care, should result in improved health [46].

While personalisation schemes are characterised by a range of different mechanisms and administrative structures, the central tenant revolves around enabling citizens to ‘purchase’ services that best meet their needs from a service market [22, 52]. While the concept of personalization is simple, the systems through which such policies are administered are hugely complex [7, 21, 44]. This complexity may in fact be a constraining factor when it comes to the ability of citizens to exercise choice and control. While personalised schemes in theory can be tailored to the specific needs of each individual, the complexity of their administration may mean that some individuals will be able to ‘work’ the system better than others and derive greater benefit as a result. Research has shown that higher socio-economic groups draw greater benefit from public services, such as education, and that this is because of the individual skills and resources at the disposal of such groups, which help them to negotiate and advocate within service systems [26, 27, 48, 49]. Moreover, research into the differential benefit derived from services by the higher socioeconomic groups [4, 13, 19] suggests that this impact may be unequally distributed across social groups. This is consistent with research into the social gradient in health [46], as well as early evidence regarding the take up of health care encapsulated by the term ‘inverse care law’ [25]. As Matthews and Hastings [48, 49] have argued, those in higher socio-economic groups may derive more health and social benefits from services because of their ability to negotiate these complex and bureaucratic service systems.

Within social welfare debates, the potential for personalisation schemes to benefit higher socio-economic groups more than lower socio-economic groups has received little investigation. In this paper, we aim to bring together existing evidence to show how top down (e.g. policy design) and bottom-up factors (e.g. individual circumstance) are intersecting in the context of one major personalisation scheme, in order to shed light on how individual social determinants of health collide with the complexity of personalisation delivery systems to entrench health inequalities. We argue that personalization schemes are in danger of embedding assumptions in their design that privilege higher socio-economic groups. If we are to ensure that personalisation schemes deliver on their promise of choice, control, and participant empowerment for all, the systems through which they are delivered need to be designed in such a way as to not privilege those already at the top end of the social gradient.

## Background

The connection between choice, control and empowerment is captured by Meagher and Goodwin [53]19:

“It’s [marketisation’s] concept of the individual as a person with rights to autonomy and participation in their personal, social and political worlds, and choice is one means through which each person can enact self-determination. The perspective within this frame is person centred: choice is a means of expressing and maintaining identity, dignity and autonomy. Self-determination or control over one’s own life is the goal, and choice enables this.”

In other areas, many have argued for ‘an ethics of care that promotes human rights’ [55], or a care and justice based ethic in the construction of systems of care [34]. Key in these approaches are individual freedoms and autonomy. While valuable on their own terms, choice, control – and the identity, dignity and autonomy they can create – are also believed to result in better outcomes for individuals [38]. These arguments are substantiated by findings from the World Health Organisation’s Commission into the Social Determinants of Health and the more recent ‘Marmot Review’. Both of these major inquiries demonstrated a link between control over ones’ lives and health outcomes [20, 46].

Personalisation schemes can be argued to be the end product of debates about choice and autonomy in care systems. Within personalisation schemes, citizens are empowered to make decisions about what services and supports best fit their needs and life. While no single model exists, personalisation puts greater emphasis on citizen choice. Funds are devolved directly to service users to purchase services from the ‘market’ (sometimes through direct transfer of funds, in other cases through voucher systems) [23, 60].

Personalisation schemes have emerged in many areas of social care, particularly disability and aged care, in countries such as the UK (C [60]), Germany [32] and Australia (Catherine [62]). These schemes emerged out of a demand from communities for more empowerment and choice, as articulated above, as well as the growth of market mechanisms in the delivery of government-funded services [39]. For governments, the use of markets (from which individuals purchase their services) were advocated on the basis of efficiency gains [39].

As noted in our introduction, there is a growing body of work which examines whether and why some social groups derive more benefit from services than others. In health, this has been dubbed the ‘inverse care law’ [25], and later extended to the ‘inverse prevention law’, after similar trends were noted in health promotion campaigns [9, 51]. These concerns sit within a broader and long running debate over how effective different welfare states are at redistributing social benefit [15, 57, 72]. Crucially, there is also evidence to suggest that access to choice and control over care is not equitably distributed [35, 67, 68], even in approaches such as personalisation, which aim to be very person-centered in their delivery.

Matthews and Hastings [26, 27, 48, 49] have argued that the middle class derive greater benefit from welfare services because of an alignment between their ‘habitus’ (a concept drawn from the work of Bourdieu [5, 6]) and welfare services. That is, welfare services emerge out of the social and cultural norms of the middle class and therefore are better tailored to the needs of those groups. Additionally, the middle class have skills and knowledge which enable them to better negotiate administrative systems and self-advocate [26, 27, 48, 49]. Personalisation schemes put an unprecedented emphasis on individuals to navigate care systems and advocate for their own needs and rights [73, 74]. As such, they pose significant potential to result in disproportionate benefits to higher socio-economic groups, entrenching or expanding social gradients in health. We examine this issue through the case of the Australian National Disability Insurance Scheme (NDIS).

### The Australian National Disability Insurance Scheme

The NDIS is Australia’s most extensive foray into personalisation. Choice and control are central tenants of the NDIS. They are both the platform on which the grassroot activists advocated and the tenants that gained bi-partisan political support for the scheme [71]. Further, they are key objectives of the NDIS Act [17], that is, to “enable people with disability to exercise choice and control in the pursuit of their goals and the planning and delivery of their supports” (Section 3e). The reform changes many aspects of the disability care system in Australia including the structure of the disability care market [7, 24, 41], systems of accountability [41, 42], and equity of access [14].

The NDIS encompasses a new financing arrangement for disability care for Australians with a permanent and significant disability, centred around principles of personalisation and individual budgets. The scheme began as a series of trails in 2013 and was nationalised in 2017. At the time of writing, the national scheme is just one year old (though some trial sites have been running for around five years). Structurally, money for care and support are allocated to each individual participant based on their needs. Each participant has their own individualised budget of Commonwealth money from which to buy services and supports from registered providers, who form a marketplace [3]. The size of each person’s budget, and the types of services it can be used to purchase, is decided annually with an NDIS planner and the person with disability, and potentially a chosen advocate (ie: family member, friend, paid advocate or other) [3].

Emerging research has suggested that choice and control is experienced differently in the NDIS depending on participants’ socio-economic context. To explore this issue and shed light on the relationship between personalisation and inequity internationally, we conducted a structured review of empirical research into the NDIS relating to equitable access.

## Methods

There has been wide-spread media coverage and internal government inquiries noting the lack of data transparency regarding the main implementation agency for the NDIS, which holds data on participants and their plans [1, 2, 31, 64, 65]. With this lack of publicly available data, we conducted a structured review of existing empirical work on the NDIS. The structured review sought to analyse the existing evidence base to determine whether different social factors put constraints to individuals’ choice and control over their care.

The search terms used were:

- (National disability insurance scheme, NDIS) AND (choice, control, empowerment, marginalisation, social determinants, health, equity, equality, gender)
- (Australia and personalis*, individualis*, disability) AND (choice, control, empowerment, marginalisation, social determinants, health, equity, equality, gender)

**Fig. 1:**
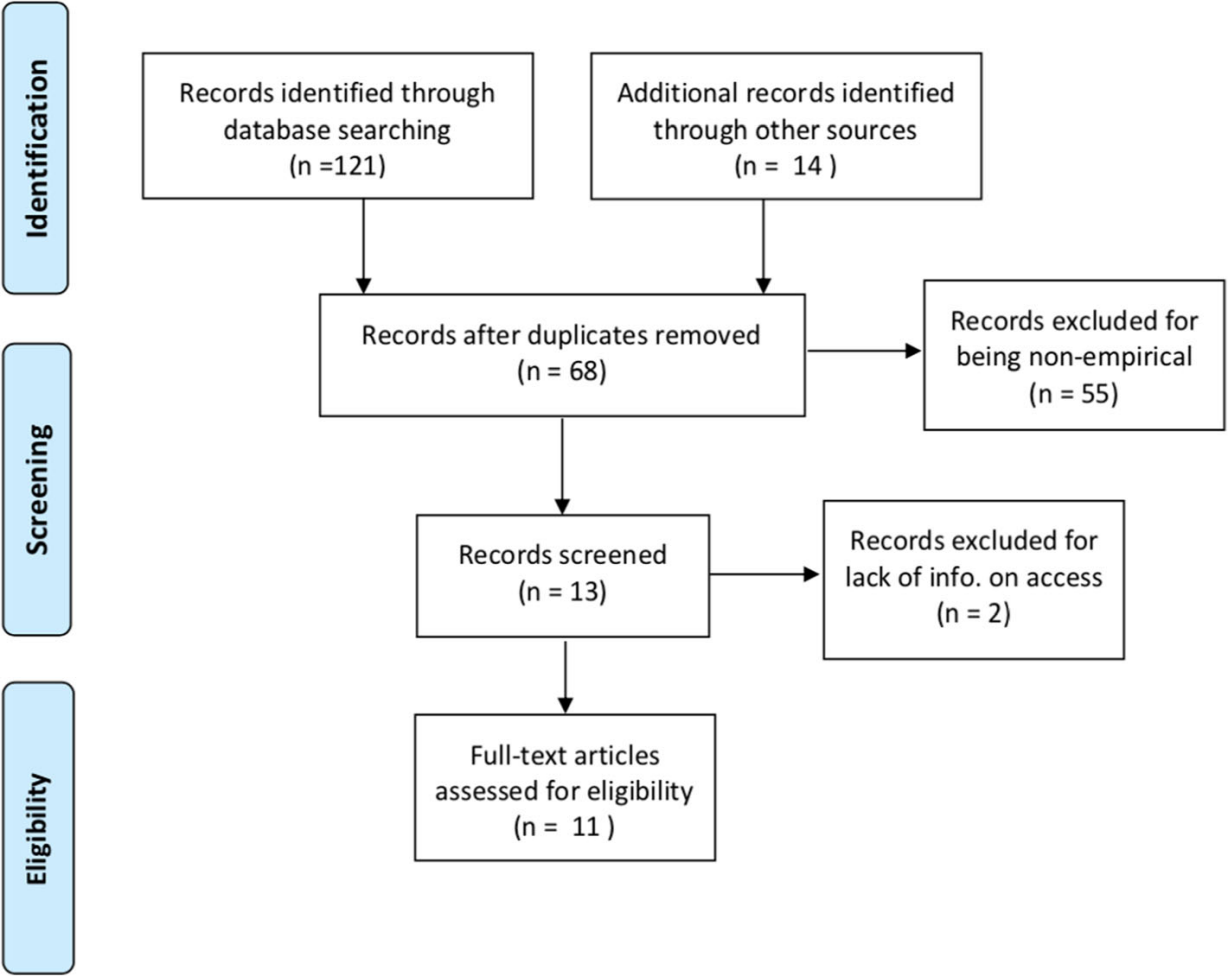
PRISMA diagram of the search strategy

The following databases were included in the search: ProQuest, Sociological Abstracts, PubMed, Web of Science, Science Citation Index, Social Sciences Citation Index, MEDLINE, Academic Onefile, ScienceDirect, Expanded Academic, EBSCO. We also scanned the reference lists of selected articles to find other useful research and included government documents from relevant government websites (i.e. those charged with the design and/or implementation of the NDIS): the National Disability Insurance Agency and the Department of Social Services, and other sites of agencies that have produced work related to the NDIS, such as the Commonwealth Ombudsman, the Productivity Commission and the National Audit Office. As the NDIS was established with the NDIS Act in [17], our timeframe was 2013 to 2018 (present). The search strategy is described in the PRIMA diagram below (Fig. 1).

The documents’ abstracts and executive summaries were reviewed by two authors to gain insight into potential constraints on choice and control. Some documents include constraints on ‘choice and control’, but do not explain them explicitly in these terms. As a result, analysis was guided by the rights-based frameworks for disability [33], and the evidence base on social determinants to health and health equity [20, 45, 46]. After reviewing abstracts and executive summaries, the sample was refined to 15 documents and articles, which provided insight into how different groups were experiencing the NDIS (in Table 1.). The criteria for inclusion were as follows:

- Research was empirical (qualitative or quantitative)
- Research focussed on NDIS in at least one case study
- Data were analysed on the basis of social or health related status, or included data that could be analysed for social factors relating to care

**Table 1:** Summary of sources, associated methods and sample sizes

	Source	Methods and sample size
1	Mavromaras et al. [50]	Surveys with participants and their families (*n* = 6246) Surveys with service providers (*n* = 2672) Qualitative interviews with participants and their families (*n* = 123) Qualitative interviews with service providers (*n* = 50) Qualitative interviews with other stakeholders (*n* = 114)
2	Warr et al. [73, 74]	Qualitative interviews with service users (*n* = 42)
3	Carey et al. (2017)	Review and analysis of government documents relating to NDIS design (*n* = 25)
4	ACT Hearing of the Joint Standing Committee for the NDIS: Market Readiness (2018)	Official transcript of proceedings (primary data)
5	Laragy et al. [37]	In-depth interviews with scheme implementers in Western Australia (n = 11)
6	Green et al. [24]	Semi-structured interviews with NDIS service providers (*n* = 29)
7	National Disability Insurance Scheme Costs: Issues paper (2017)	Review of NDIA annual and quarterly reports (sample size not provided)
8	Ombudsman’s report (2018)	Official review of complaints about the NDIA (approx. *n* = 1200), stakeholder feedback and briefings provided by the NDIA
9	Meltzer et al. [54]	Two forums with providers and community members linked to service provision (*n* = 19 and *n* = 64 respectively) Telephone interviews with early childhood intervention providers and related providers (no sample size provided)
10	Cortis et al. [18]	Survey of disability support workers (*n* = 1476) Survey of CEOs of not-for-profit organisations registered to provide NDIS services in NSW (*n* = 135) Interviews with disability support providers in NSW (*n* = 20) Review of NDIS pricing documents
11	Lakhani et al. [36]	Interviews with people with disability and their guardian(s), family member(s), and/or carer(s) in South-East Queensland (*n* = 70)
12	Purcal et al. [66]	Interviews with family members and service providers of children in the NDIS in the Hunter region (*n* = 38) Surveys with family members and service providers of children in the NDIS in the Hunter region (*n* = 344)
13	Henekar et al. (2017)	Interviews and focus groups with service providers, NDIS participants, non-NDIS participants with disability, and community members (*n* = 55)
14	Hui et al. [29]	Interviews with low SES people with disability about access to NDIS in Woolongong (*n* = 32)
15	Collings et al. [16]	Focus groups with planning practitioners in New South Wales (*n* = 99)

The 15 documents were analysed thematically for references to social factors affecting care: gender, socio-economic position, education, geographical location, culturally and linguistically diverse groups. Through this analysis we identified a range of factors influencing choice and control within the scheme (outlined in the first part of the results section below). We then focused our analysis on how the administrative structures and systems of the NDIS intersect with individual circumstances – advantaging some, while disadvantaging others. This was derived from evidence in the documents reviewed, combined with our research on administrative structures in the NDIS [11, 12, 41, 43]; Eleanor Malbon, Carey, & [21]).

Notably, a major source for this review is Mavromaras et al. [50] is a large scale evaluation of trial sites of the NDIS, from which we draw interview quotes and statistics. The trials included a mixture of whole populations and trials targeted for specific age groups, including early intervention and school leavers. The design of the administrative systems in trial sites mirror that of the full scheme and the evaluation is indicative of how the national scheme functions.

## Results

In our thematic analysis, we establish that the NDIS systems intersect with individual circumstances to constrain or enable choice and control. Within this overall finding, we will discuss 1) individual budget management 2) dependence on market robustness, 3) bureaucratic accessibility, and 4) service provision. Prior to exploring these themes, we review the direct evidence that different groups are benefiting disproportionately from the scheme.

Our review revealed disproportionate benefits and difficulties for some groups accessing the scheme [29, 36, 37, 73, 74]. For example, the trial evaluation and other reports found that ‘vulnerable groups’ are less likely to receive funded supports than other NDIS participants with similar needs [50]. The evaluation details some of the conditions that can constrain opportunities for choice and control for personalisation scheme participants:

“Those more vulnerable to poorer outcomes included participants with intellectual disability and/or complex needs; from CALD [culturally and linguistically diverse background] communities; those experiencing mental health, substance abuse, or forensic issues; and older carers who were socially isolated and had their own health issues. These vulnerable groups were considered to receive less funded supports in their NDIS plans than others with similar support needs and to struggle with NDIS processes.” ([50]:199).

Further to the explicit identification of ‘vulnerable groups’, the evaluation of trials also identified groups that benefit most from the NDIS, [50]. Though precise figures were not provided, the following quote identifies that males and people with higher income are more likely to find a service provider to fulfil their care requirements:

“ … male participants and those with a higher household income were shown to be less likely to experience unmet demand for supports” ([50]:23).

An assessment of the design of the NDIS (Carey et al. 2017) proposed that differences in disability type, remoteness of living, age, gender and access to market are all likely to result in inequitable uptake of the scheme. Similarly, a series of in-depth interviews about the NDIS in Western Australia highlighted that there is nothing ‘automatic’ about a personalisation scheme that leads to greater choice and control [37]. They highlight that people’s circumstances enabled or constrained their ability to act on choice:

“individualised funding packages did not automatically result in more choice and greater opportunities. People needed information to make informed decisions; supportive and creative support from social workers and other professionals; and welcoming communities” ([37]:282).

This finding is echoed in Meltzer et al.’s [54] report into early childhood interventions in the Hunter region, and also noted by a recent study into the experiences of the NDIS by participants [73, 74]:

“Factors that are well-recognised in driving inequality – household income, education, residential location and household structure – remain critical in filtering opportunities and capacities for service users and their carers to have choice and control in accessing services and resources under the NDIS.” [73, 74]:9.

### Individual budget management by participants

Each NDIS participant has their own individualised budget, and these can be administered by the participant (‘self-managed’), chosen by the participant but administrated through a plan manager who pays invoices on behalf of the participant (‘plan-managed’), NDIA managed, or a combination of these. Self-managed participants make up 7% of NDIS participants [58]. Self-managed participants carry a higher administrative burden themselves [73, 74], however, they are more straightforward clients for service providers, as they pay the service provider directly without the service provider dealing with the NDIA and allowing service providers to negotiate their own prices [59]. This means that self-managed participants are most able to negotiate for more tailored, boutique services and innovations:

“We now only work with self-managing and plan-managing participants and charge our own rate (not the NDIA rate).” ([59]:50).

However, the people most able to self-manage are likely to be advantaged in other ways, and theory tells us, are more likely to be upper or middle class users [48, 49]. Concerningly, research on people with intellectual disabilities in the scheme found that up to 40% of participants believe they require further training to fulfil the administrative tasks of the NDIS, including looking after money, working on computers, finding the right service for the right price, talking and writing, planning time, making choices and being heard ([36]:795).

Inequities are also apparent at the point of the planning meeting, where the individual budget is decided upon. The early stages of the NDIS have been characterised by inconsistencies in individual budgets between people with similar needs, who might be expected to receive similar sized care packages [64, 65]. As an example of what this looks like, the parent of a child in the NDIS early intervention trial observed that:

“Apparently my plan should only be about $12,000... That’s completely generous apparently, the $12,000. I am getting $18,500.” ([50]:96).

The NDIA has observed these inconsistencies in their annual reports, and claim that variability in NDIA planners is a reason for this:

“There is greater than expected variability in package costs for participants with similar conditions and levels of function (suggesting inconsistencies in planners’ decisions).” ([64, 65]:10).

However, inconsistencies between individual planners are not the entire story when it comes to differences in individual budget management. The NDIS trial evaluation [50] and report by Warr, Dickinson, Olney, Karanikolas, Kasidis, Katsikis, and Wilcox [73, 74] both provide compelling evidence to suggest that participants with stronger supports around them during plan negotiation may have plan budgets that are larger than others who are less enabled or practiced at negotiation. A service provider explains the difference that a knowledgeable advocate can make in the planning meeting:

“We had a carer come in whose wife had younger onset dementia … his wife’s initial plan was $700. Their second plan was $600, and when they had a review of the plan with the assistance of a key worker they were able to get nine hours of home care and a week of full respite with 24/7 care. That jumped to $32,000. I think that is a real great snapshot of the difference of having someone to come in, advocate and really also prepare the person for their meeting.” ([50]:248).

As advocacy is not funded in the NDIS, it often falls to families to use their own skills be advocates or resources to fund advocates, leading to potential inequities in access to advocacy services:

“The NDIS was considered to work best for participants and families who were able to strongly advocate for themselves. In order to ensure equitable access to funding for all participants, the importance of advocacy (either formal or informal) was highlighted. Concerns were raised, however, about a lack of funding for formal advocacy support under the NDIS.” ([50]:185).

Warr et al. (2017:47) explains how middle-class participants and family members can negotiate the planning process with greater ease:

“Participants and parents who could draw on professional experience which gave them an understanding of the logics of meetings, preparing funding requests and liaising with professionals, appeared to be more confident and assertive in their interactions in planning processes, compared to participants who had previously had limited exposure to these kinds of processes.”

### Dependence on market robustness

The structure of the NDIS as a personalisation scheme means that choice and control of services is dependent on market robustness [3]. Without a well-functioning market, multiple providers are not available for participants to choose from. The success of the NDIS relies upon participants being able to exercise choice and control in the selection of their care services [3] and for this to occur there needs to be multiple and good quality suppliers in the market, and participants also have to be empowered to make choices and change when providers are inadequate or undesirable (Catherine [61]).

A hallmark of a poorly functioning personalisation market is a ‘thin market’, which occurs when there are zero or very few providers of a certain service in a local area, or if the available service providers are full and cannot take on more participants [7]. This might also be referred to as a market failure or a market gap. Awareness of the problem of thin markets in the NDIS has been present since the early days of implementation [7, 56] and is increasingly seen as a pressing problem (Carey et al. 2017). Like many constraints to choice and control, this is a structural problem which is felt unevenly or inequitably across the population of individuals with access to the NDIS. For example, remote and regional areas may be more prone to thin markets due to the potentially vast distances between participants and providers. Or, some particular services, like those that service people with psycho-social disability and/or challenging behaviours, may be under serviced due to the difficulties present in providing those services.

Further entrenching disadvantage for people who experience thin markets, spending restrictions in the NDIS means that money in NDIS plans that is unspent by participants may be reclaimed by the NDIS:

“Currently, the status quo is that … ‘You didn’t use X amount of dollars in your plan, so therefore you lose it,’ and no-one’s (a) monitoring the fact that they couldn’t access services and they’ve been sitting on a waiting list for two years of their plan or (b) taking any steps to help that participant to retain that funding in the hope that, as they move forward, those services may become available.” ([30]:9).

The evaluations of trial sites found that the people least likely to find providers for their care are women, non-men and people with lower education levels [50]. This suggests that such groups are more likely to have their care funds reclaimed by the NDIA, further entrenching inequitable divides.

Along with thin markets, changes to competition in the NDIS markets also impact on choice and control by changing the dynamics between service providers and people with disability [24]. While the main purpose of the NDIS is to change relationships between service providers and people with disability [71], this was specifically to give more empowerment to people with disability. Instead, through the marketisation of the NDIS, some service providers have reduced the flexibility and availability of care that they previously supplied. A participant in the trial evaluation observed that:

“I noticed quite a shift in service providers attitudes that bothers me, that the service providers, even ones that we’ve dealt with for quite a time, who were very flexible and very helpful, and really treated us as part of the family... they’re so fed up with it that they really are getting like ‘No I won’t, not unless they pay.’” ([50]:67).

Service providers have also reported that they are less able to respond to crisis events in the general population, as the new structure of personalisation means that they cannot be paid to help someone unless that person has money in their plan for that specific service. In a report on changes in the sector, this service provider explains:

“At the moment, someone rings us on a Friday afternoon and says they’ve got a crisis for a client we’re at liberty to say no dollars, no interest, aren’t we?... if they’re not our clients we haven’t got their package, we haven’t got any hours of coordination for them …. Under the old system if we get phone calls from the police and say ‘so and so’ was found wandering the street, can we do something? You know we’d send one our case managers out, we might do all sorts of things, but that was just because we were funded to do this sort of stuff across the community. But under the new model if we ain’t got an hour of coordination for a person I can’t allocate an hour staff time.” ([24]:18).

As in the quote above, inflexibility of services and constricted availability of crisis support may be felt inequitably between participants of the NDIS, with people who are less able to secure a regular and flexible service provider missing out on crisis support and services specifically tailored to their needs, or potentially not able to qualify for the scheme at all. Participants vulnerable to this include people with complex mental illness and challenging behaviours.

### Bureaucratic accessibility

A further factor influencing individuals’ choice and control is levels of knowledge and understanding about navigating the NDIS, it’s bureaucratic accessibility. This is particularly the case should participants choose to self-manage their funding (the highest form of choice and control). Participants who self-manage take care of all administration related to their care and supports, rather than using a third party. However, even when a third party is used in the coordination of their plan, participants are required to understand the details of the scheme’s administration – including the planning process, offer and take up of services, and use of scheme resources, such as a complex online NDIS payment portal – in order to make the most of their decisions and choices about their care. This implies the need for participants (or their nominees) to have the skills and time to understand rules, intent, infrastructure and the operational details of the scheme.

A recent transcript from a Senate inquiry into the scheme suggests that such skills do not always come easily to participants:

“I think it’s worth noting that the ability of people to get the outcome that they want really depends on their skills in navigating bureaucracy and being able to do those wily things. In which case, we’re likely to see these kind of stratified outcomes from the scheme depending on what kinds of skills people have.” ([30]:34).

Having the skills to navigate the system appears to be mediated by a range of social factors, including cultural and language background, literacy level and level of complexity of need, with people who have compounding experiences of disadvantage or trauma often experiencing difficulty finding their way through systems [16, 28, 29]. For many of these groups, accessible information about systems is of critical importance, and the availability of such information has been recognised as a key component of consumer rights [40].

While the NDIS does have some accessible (or ‘Easy Read’/‘Easy English’) information available, there are still reports of the system being difficult to understand and navigate, including those who describe it as “protracted, unpredictable and intensive” especially for people with complex needs ([16]:149). In particular, there are reports of the difficulties presented by information availability and frequent changes bureaucratic processes (likely caused by pressure to roll-out the scheme quickly), which even sector staff may not always understand themselves. Two parents of young children entering the NDIS for the first time noted this difficulty:

“What worries me is how it’s constantly changing all the time ... it’s one thing today and then tomorrow might be something different.” ([66]: 14).

“Every time I have called the NDIA and spoken to health professionals about it, I get a different story. No one seems to know what is going on, and I keep getting palmed about and not receiving callbacks as promised by NDIA.” ([66]: 16).

In addition to the complexity of information, specific implementation issues in the scheme have also affected the level of bureaucratic accessibility of the NDIS and capacity of participants to exercise choice and control. The NDIS online portal to view and access details about one’s plan and funding expenditure has been a key issue [1, 2]. The lack of intuitiveness of the portal, requirement for internet access and digital literacy are some key problems, impacting particularly those for whom digital access is a challenge:

“Issues with the portal were particularly prevalent in low-income households and we spoke to many participants, particularly those with cognitive disabilities and older parent-carers, who had limited or no access to mobile phones, other devices or the internet.” [73, 74]:38.

Further, there was also a collapse of the technical infrastructure of the portal, which caused it to be ‘down’ for a period of time, preventing access and confusing scheme participants. A lack of availability of assistance from the NDIA with navigating the portal is also a reported issue that compounded this problem:

“I’d keep ringing [the NDIA] yet was shoved around from this person to that person. ... I think if everyone was assigned to someone... and you can ring them and they have the ability to help you directly.” ([66]: 22).

Bureaucratic accessibility problems means that many people have reported relying on personal networks and peer support systems to complement their understanding of the NDIS and receive information about the scheme from people they trust [29, 54, 66]. Others have emphasised the importance of being connected to good local service providers who can explain key details to them (Purcal, Hill, Meltzer, & Fisher, 2018). The challenge of these solutions in terms of the social determinants of health and for people from low socio-economic backgrounds is that they rely on a high level of social capital and access to effective service providers, and those who are socially isolated or otherwise disadvantaged may not have access to these forms of assistance. Thus, the bureaucratic nature of the scheme and challenges for accessibility – including the dependence of help on one’s own networks – means that choice and control may be limited for those who are socially isolated or lack skills and resources to navigate complex service systems.

### Service provision

Changes in the way that service provider staff are expected to work within the NDIS and in how they are paid and managed can also have flow on effects for scheme participants. In particular, constraints on and changes in the practices and operation of service providers have the potential to impact on choice and control for participants. This is especially the case for those with complex needs and/or those who experience barriers in many intersecting social determinants of health.

The shift to individualised funding – while a fundamental tenant of personalisation policies – represents changes for care and support staff in how they are expected to operate. One challenge in the scheme is in funding the training and wages needed to maintain high quality staff:

“Low NDIS prices are causing staff to be employed on lower wages, making it difficult to attract and retain quality staff. This will lead to [a] decrease in quality services provided to people with disability. Staff will receive less training to the detriment [of] people with disability.” ([18]:16).

Other challenges are having enough funded hours within an individualised funding packages to facilitate high quality and coordinated service provision, such as spending time getting to know participants and/or collaborating with other service providers who may also be working with a participant:

“Whereas previously there’s been a lot of time to work with people in a more person-centred way, get to really know them, what their goals are, how we’re going to help them, and support them to achieve those goals. … (Now) (t) here are a lot of participants out there that you feel really concerned about because their family may not have capacity to provide for their disability support needs, and you’re working [in] a really complex system I suppose where there’s lots of different isolated systems, trying to work together to support somebody and it doesn’t always work very well.” (Cortis, Macdonald, Davidson, & Bentham, 2017:12).

“Prices do not account for what is required to deliver high quality services, and arrangements are not fully enabling disability support workers to deliver services which are personalised, co-ordinated, responsive or safe. Quality is likely to diminish in the process of NDIS expansion.” ([18]:1).

While research into the transition to the NDIS suggests that some service providers are finding new ways to fund collaboration [54], it remains a challenge, with potential impacts on the quality of services provided to NDIS participants and hence on the level of choice and control they can enact. As participants who experience complex support needs and/or many compounding barriers in terms of social determinants of health typically access more services and may need more coordination among the variety of service providers who assist them (Collings et al., 2015), this challenge has the potential to disproportionately affect this group and constrain their choice more than their middle class peers, who may not have as many service providers in their lives.

Further, while choice and control are meant to sit with participants in the NDIS, due to the introduction of a service marketplace, service providers and individual workers also have greater capacity to determine which clients they are willing to work with. Reports indicate that some service providers are choosing only self-managed NDIS participants [24, 59]. This disadvantages those unable to or do not want to self-manage their funding. In addition, some individual workers may be reluctant to work with the most complex clients, which can affect those clients’ capacity to enact choice and control between services, even if they have funding available:

“People talk about us having choice and control but … They’ve got individual workers saying, ‘No, I don’t like that client, that client’s got behavioural problems, I’m not working with them’. So they’ve got individual workers that are now picking and choosing their clients. So you’ve got clients with the most complex needs … they can’t find support workers …” ([73, 74]:49).

The impact of these changes – what some have called the “Uberisation of the sector” ([73, 74]: 68) – is that scheme participants with the most complex needs may be disadvantaged in enacting choice and control. Where there are more coordination costs and where service providers and workers may choose not to work with them, people with disability and complex needs may not have the same choices for services as their middle-class peers [29]. In this respect, the new context for service providers is another constraint on the operation of choice and control, which intersects with the social determinants of health for many people with disability.

## Discussion

While there is a growing body of work exploring how and why higher socio-economic groups derive greater benefit from government services [26, 27, 48, 49], it has been ignored in the context of personalisation schemes. This is concerning on two levels. Firstly, personalisation is growing in the provision of social care in many countries and we currently do not know how it impacts inequality. Secondly, there are reasons to believe personalisation may have a stronger ‘inverse care law’ than other services, as such schemes put an unprecedented emphasis on individuals to navigate care systems and advocate for their own needs and rights. As one of the most ambitious personalisation schemes in the world [43]), the NDIS provides an important case through which to examine these issues.

Our review of the existing empirical research and evaluations of the NDIS supports the argument that the structure of administrative systems within personalisation schemes favor those already equipped to deal with complex bureaucracy (counter to the claims of choice, control and empowerment). We find that the NDIS has a number of structural aspects that can result in inequitable access to the scheme or to care services, with flow on effects for choice and control, empowerment and health outcomes. From this we conclude that the NDIS, and personalisation schemes more broadly, still privilege a vision of the “competent” and “independent” person who can take on the additional administrative and decision-making burdens. This aligns with previous research. For example, Matthews and Hastings [48, 49] argue that middle-class users are more favored in the design of public services because those designing and administering public services are also likely to be middle-class, resulting in services that match the values and norms of the middle-class. In other words, services are created with a particular norm or ideal user in mind and these reflect the designers themselves.

With regard to personalisation schemes, our findings suggest that such approaches have the potential to entrench existing inequalities. We found evidence of inequitable access occurring along the lines of gender [50], education [50] remoteness and rurality (Carey, [43, 50, 73, 74]), socio-economic position [29] and disability type [36, 50]. As presented in the findings, there are structural aspects of the delivery systems of personalisation schemes that favour users who have good literacy, speak English, hold low levels of trauma, trust systems, haves the time to manage their own funding and to research the choices available, or have a trusted person to do this for them, and so on. In other words, these are people who are likely to already be situated near the top end of the social gradient of health [47] and have high social capital. These attributes and social conditions can negatively interact with administrative systems for personalisation – highlighting the need for more consideration of social and health inequailities during design and implementation [10]. Olney and Dickinson note, administrative burden in personalisation is distributed unequally [63]. In the context of the NDIS, we found that this was likely to occur in four key areas: managing individual budgets, bureaucratic accessibility, service provision and market robustness. These are defining characteristics of personalisation schemes internationally [60], suggesting that such schemes have the potential to entrench and widen social inequalities by nature of their very design.

Despite attempts to increase choice and control in personalisation approaches, these programs can none-the-less remain inflexible to many individuals’ circumstances and needs. While personalisation schemes such as the NDIS cannot necessarily redress existing inequities in the social determinants of health (e.g. location, differential levels of education), in theory they should at least not widen or perpetuate these inequities and should provide additional support to those who are disadvantaged when using their systems. Given the focus on choice, control and empowerment, a well administered personalisation scheme with thorough supports could result in some levelling of the social gradient – enabling citizens to access services and supports that meet their needs without widening inequities. However, the systems through which ‘personalisation’ is delivered may still be developed with an ideal norm in mind, which does not account for variations across the population. This is not a fundamental flaw in personalisation itself, but rather something to consider in the design and delivery of personalisation schemes, which may or may not entrench inequities depending on how they are designed and administered. As a result, personalisation may not only entrench existing inequities, but widen them by allowing those higher on the social gradient to derive more benefit than those situated lower on the social gradient. Experiences of the NDIS suggest that this very possible. Such findings have widespread implications for efforts to ‘flatten’ the social gradient in health.

## Conclusion

Despite the considerable growth in personalisation schemes in disability and aged care internationally, to date little research has examined their effects on social inequalities. On the one hand, we might hypothesise that with their emphasis on choice, control and empowerment, personalisation schemes have the potential to address individual differences in social determinants to health, leading to greater equity. However, such schemes put unprecedented emphasis on individuals to advocate for their own rights and navigate burdensome administrative systems. In examining one of the largest and most ambitious personalisation schemes in the world, the NDIS, we found evidence that the very design of these schemes can not only entrench existing inequalities in the social determinants of health but widen them. This is concerning given the international push towards personalisation in various areas of social care, with widespread implications for efforts to address the social gradient in health. More attention needs to be given to the administrative structures and systems through which personalisation schemes are delivered if we are to avoid increasing inequity.

## Data Availability

All data is contained within the manuscript file.

## Abbreviations

LACs: Local Area Coordinator
NDIS: National Disability Insurance Scheme
